# Hypothalamic oxidative stress and inflammation, and peripheral glucose homeostasis in Sprague-Dawley rat offspring exposed to maternal and postnatal chocolate and soft drink

**DOI:** 10.1038/s41387-018-0051-z

**Published:** 2018-07-19

**Authors:** Marina Kjaergaard, Cecilia Nilsson, Mette Olaf Nielsen, Kevin Grove, Kirsten Raun

**Affiliations:** 1 Diabetes and Obesity Research, Novo Nordisk A/S, Måløv, 2760 Denmark; 2 Department of Veterinary and Animal Sciences, Faculty of Health and Medical Sciences, University of Copenhagen, Frederiksberg C, 1870 Denmark; 3 Uppsala University Innovation, Uppsala Science Park, Uppsala, 751 83 Sweden; 4 Division of Diabetes, Obesity and Metabolism and Division of Reproductive and Developmental Sciences, Oregon National Primate Research Centre, Oregon Health & Science University, Beaverton, OR 97006 USA

## Abstract

**Background:**

Predisposition to obesity and type 2 diabetes can arise during foetal development and in early postnatal life caused by imbalances in maternal nutritional overload. We aimed to investigate the effects of maternal and postnatal intake of chocolate and soft drink on hypothalamic anti-oxidative stress markers, inflammation and peripheral glucose homeostasis.

**Methods:**

Pregnant Sprague-Dawley rats were fed ad libitum chow diet only (C) or with chocolate and high sucrose soft drink supplements (S). At birth, litter size was adjusted into 10 male offspring per dam. After weaning at 3 weeks of age, offspring from both dietary groups were assigned to either S or C diet, giving four groups until the end of the experiment at 26 weeks of age.

**Results:**

Offspring exposed to maternal S had up-regulated hypothalamic anti-oxidative markers such as SOD2 and catalase at 3 weeks of age as an indication of oxidative stress. However, at 12 weeks of age these anti-oxidative markers tended to decrease while pro-inflammatory markers such as TNF and IL-1β became up-regulated of all offspring exposed to S diet during some point of their life. Thus, despite an increase in anti-oxidative stress response, offspring exposed to maternal S had a reduced ability to counteract hypothalamic inflammation. At the same time point, postnatal S resulted in increased adiposity, reduced glucose tolerance and insulin sensitivity with no effect on body weight. However, at 25 weeks of age, the impaired glucose tolerance was reversible to the response of the control regardless of increased adiposity and body weight pointing towards a compensatory response of the insulin sensitivity or insulin secretion.

**Conclusion:**

Indications of hypothalamic oxidative stress was observed prior to the inflammatory response in offspring exposed to maternal S. Both maternal and postnatal S induced hypothalamic inflammation prior to increased weight gain and thus contributing to obese phenotype.

## Introduction

Obesity is an important risk factor for metabolic diseases such as Type 2 Diabetes and cardiovascular diseases, and is associated with higher rates of mortality^1^. The primary cause of obesity is when food intake exceeds energy expenditure. Today’s abundance and easy access of palatable diets and snacks rich in fat and sugars contribute to the problem^2,3^. Consequently, many pregnant women have a substantial intake of fat- and sugar-rich diets which can affect the foetal development and predispose progeny to obesity^4,5^. Increased nutrient supply to the foetus can alter the development of neuronal circuits of the brain^6–10^ as well as other essential organs^11^ that are critical to regulation of growth and energy homeostasis^4,5^.

The hypothalamus is the predominant brain area that controls energy balance. Growing evidence suggests that the hypothalamus is one of the first tissues to show signs of impairments upon high fat diet (HFD) exposure. For instance, HFD has been shown to induce hypothalamic inflammatory responses resulting in a dysfunctional regulation of energy balance^12–14^ and glucose homeostasis^15–19^. There are several suggested mechanisms behind diet-induced inflammation including mitochondrial dysfunction, reactive oxygen species (ROS), and endoplasmic reticulum (ER) stress associated with unfolded protein response^20–22^. Oxidative stress is defined as a persistent imbalance between the production of ROS and the naturally occurring antioxidant defence mechanism^23^.

Enzymes as catalase and the two anti-oxidative superoxide dismutases (SODs) are considered to be the first line of defence mechanism and play an important role against oxidative stress^24^. SOD1 and SOD2 are exclusively expressed in the intracellular cytoplasmic spaces and in the mitochondrial spaces, respectively^24^. When ROS homeostasis is disrupted, excessive ROS, which is highly reactive to lipids, proteins and nuclei acids, are accumulated in the mitochondria and cytoplasm. This may cause cellular damage and/or inflammation, thus leading to tissue impairment and diseases^15,21,23,25^. For instance, excessive ROS in response to HFD have been shown to induce inflammation and neuronal cell death^26^, contributing to development of obesity^12^ and neurodegenerative diseases^18,26,27^. In the view of inflammation, cellular oxidative stress may activate the nuclear factor-κB (NFκB) and c-Jun N-terminal kinase (JNK) pathway which are the most well characterized cellular inflammatory pathways^15,18,21,25^. Also, the pro-inflammatory marker tumor necrosis factor alpha (TNFα) released from non-neuronal cell types such as microglia contributes to induction of JNK and NF-κB pathways^28^.

Only limited focus has been given to development of hypothalamic oxidative stress and inflammation during foetal and postnatal life. Maternal obesity and a maternal HFD appear to be risk factors for induction of early onset of hypothalamic inflammation in the offspring. In a mice study, offspring exposed to maternal high-fat diet (HFD) had increased activation of several hypothalamic inflammatory pathways such as IKKβ and JNK1 associated with obesity and reduced glucose tolerance in offspring aged 3 weeks^15^. Other studies have shown increased hypothalamic IL-1β expression in rat offspring as a consequence of maternal obesity^29^ and in non-human primate foetuses as a consequence of maternal HFD^30^.

In the present study, we investigated the effects of maternal and postnatal chocolate and soft drink supplementation (S) on hypothalamic anti-oxidative stress markers and inflammation, adiposity and glucose tolerance in rat offspring. To evaluate hypothalamic oxidative stress and inflammation, we measured real-time PCR mRNA expression of anti-oxidative stress and inflammatory markers in hypothalamus extract from offspring at 3 and 12 weeks of age. Furthermore, assessment of glucose homeostasis was performed by an oral glucose tolerance test (OGTT) at 12 and 25 weeks of age in the offspring.

## Materials and methods

### Study design

**Fig. 1:**
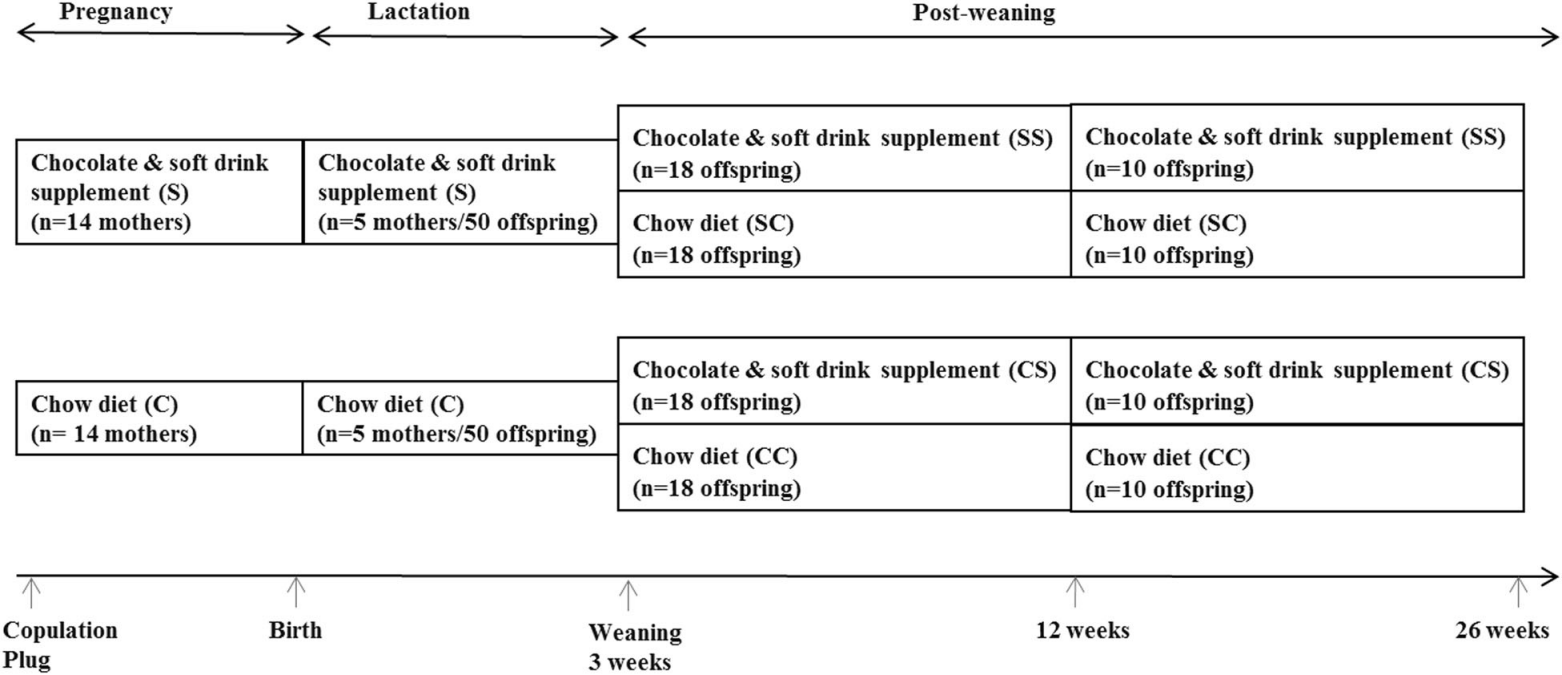
The rat study design. Twenty-eight pregnant Sprague-Dawley rats were either fed ad libitum chow diet only (C) or chow with chocolate and high sucrose soft drink supplement (S) from one day after copulation plug and until weaning of offspring 3 weeks after parturition. Litter size was adjusted into 10 male offspring per mother. After weaning, offspring from both dietary groups were assigned to either S or C diet, giving four groups until the end of the experiment at 26 weeks of age, namely CC, CS, SC, and SS. For details of animals and procedures, see Experimental materials and methods (ref.^32^)

Data in the present manuscript arise from a previously published study. Figure 1 illustrates the study design (for further details see ref.^32^). For the present publication, hypothalamic samples for gene expression analysis were collected from the offspring upon euthanisation with isofluran followed by decapitation at the different developmental stages at 3 weeks (*n* = 10/group) and 12 weeks of age (*n* = 8/group). Blood was collected at 3, 12 and 25 weeks of age (*n* = 10/group).

### Diets and food intake

All rats had free access to water from drinking nipples and were fed a standard laboratory chow diet ad libitum (Altromin 1320, Brogaarden, Denmark). The chow diet contained a caloric density of 2.8 kcal/g (24% protein, 11% fat, and 65% carbohydrates). The high-fat/high-sucrose supplementary diet consisted of various chocolate bars on an average caloric density of approximately 5.4 kcal/g (8% protein, 33% fat, 59% carbohydrates of which 53% sucrose is present). The soft drink was produced according to European standards. It consisted of a caloric density of on average 1.96 kcal/ml (0% protein, 0% fat, 100% sucrose), and was given ad libitum in bottles (at least 80 ml per day) and changed every third day. Chocolate and soft drink intake were recorded by manual weighing once a week. The maternal intake of chocolate and soft drink were estimated to be approximately 40 kcal/week during gestation and 50 kcal/week during lactation. Thus, the intake of chocolate and soft drink was 20 and 15–20% out of the total caloric intake, respectively (for further details, see ref.^31^). For each offspring, chow and chocolate intake were recorded by manual weighing once a day during five days at 11 and 24 weeks of age. The energy intake was calculated in kcal ingested daily per rat and presented as daily energy consumption per group. For further details, see ref.^32^.

### Body composition

Body composition was determined by the Echo Medical System per rat QMR scanner (EchoMRI 2004, Houston, USA) at 3, 12 and 25 weeks of age, as previously described in ref.^31^.

### Oral glucose tolerance test (OGTT)

OGTT was performed at 12 and 25 weeks of age in the offspring. Before subjecting the rats to the OGTT, offspring were semi-fasted overnight, only receiving 10 g of chow diet overnight. Chocolate and soft drink were also removed as well as clean cages were given to the rats. The semi-fasting condition was done to induce a mild degree of fasting in the rats with access to 50% of their daily energy requirements for 16 h before the OGTT. The offspring were given an oral glucose bolus (500 mg glucose/ml) of 2 g glucose/kg body weight (BW) at 12 weeks of age, and a dose of 4 g glucose/kg BW at 25 weeks of age. Blood for glucose and insulin measurements was sampled at specific time points (*t* = −2 (baseline) and at 15, 30, 60, 120, 180 and 240 min after glucose bolus). Blood for corticosterone determination was sampled 15 min after glucose administration. All blood samples were collected from the tongue vein. Calculation of the areas under the curve (AUC) for plasma concentration of glucose and insulin were evaluated to define glucose tolerance. Plasma concentration of corticosterone was evaluated 15 min after oral glucose load as a measure for stress-responsiveness of the animals.

### Blood analysis

Blood samples used for glucose, insulin, plasma triglycerides (TG), and free fatty acids (FFA) determination were analysed as previously described^31^.

### RNA extraction and real time PCR

Cellular RNA and PCR reactions were performed on the whole rat hypothalamus at 3 and 12 weeks of age as previously described in ref.^31^. The target genes were tumor necrosis factor alpha (TNFα) (Rn99999017_m1), interleukin 1 beta (IL-1β) (Rn00580432_m1), Nuclear Factor-κB (NFκB) (Rn01399566_m1), superoxide dismutase 1 (SOD1) (Rn01477289_m1), superoxide dismutase 2 (SOD2) (Rn00690588_g1), catalase (Rn00560930_m1) and18S ribosomal RNA (Mm04277571_s1) (Applied Biosystems). Level of expression of each target gene of interest was presented as a percentage of the expression of the appropriate housekeeping gene 18S ribosomal RNA. 18S ribosomal RNA was chosen as the most appropriate reference gene for the hypothalamus as it was not affected by the treatments.

### Statistics

The statistical analysis was performed in the statistical program R. Data were performed by fitting data as mixed linear models (“lme”) and analysed either as one or two factor study designs (ANOVA). The fixed factors “Maternal diet”, “offspring diet”, and “dam lactation” were used as a random factor. All variables were visually assessed and statistically tested by “Shapiro-Wilk normality test” for normal distribution. Also, homogeneity of variance were assessed by visual inspection of residual plot and tested through “Bartlett test of homogeneity of variances”. The replicate of the statistical analysis was referred to individual offspring in each group. Data are presented as means ± standard errors of mean (SEM). A *p*-value of 0.05 or less was considered statistically significant.

## Results

### Pregnant and lactating mothers

Maternal body weight and percentage of body fat mass was not affected by the S-diet during gestation. However, by the end of lactation percentage of body fat mass was increased in S fed mothers (*p* < 0.01) but there were no effect on body weight. No effect was observed on blood glucose, plasma insulin, TG and FFA levels during gestation and lactation (data not shown). Therefore, the possible changes in foetus development were a direct effect of the maternal intake of the S-diet. Furthermore, pregnancy length, litter size and offspring birth weight did not differ between C- and S-diet fed mothers (data not shown).

### Body weight, fat mass and plasma lipids

At weaning (3 weeks of age), no differences in body weight, TG and FFA were observed between the offspring groups (Table 1). However, the percentage of body fat mass was increased in offspring from S-fed mothers (Table 1). At 12 weeks of age, still no significant difference in body weight was observed between treatment groups, but the postnatal S diet resulted in increased body fat mass and plasma FFA in CS and SS compared to CC and SC offspring, and in SC compared to CC (*p* < 0.05) (Table 1). Furthermore, plasma TG was increased in SS compared to CC (Table 1). At 25 weeks of age, postnatal S diet resulted in increased body weight, body fat mass and plasma lipids of TG and FFA in CS and SS offspring compared to SC and CC offspring fed postnatal chow (Table 1).

**Table 1:** Body weight, body fat and plasma lipids (TG & FFA) at 3, 12 and 25 weeks of age

	Body weight (g)	Body fat (% fat/BW)	Plasma TG (mmol/L)	Plasma FFA (mmol/L)
Week 3				
C	45.5 ± 0.5	10.0 ± 0.5	1.7 ± 0.1	109 ± 15
S	44.4 ± 0.7	15.9 ± 0.5^***^	1.4 ± 0.1	140 ± 15
Week 12				
CC	386 ± 6	11.9 ± 0.4	0.9 ± 0.1	88 ± 7
CS	389 ± 7	16.0 ± 0.6^a***b*^	1.1 ± 0.1	266 ± 36^a**^
SC	374 ± 7	13.6 ± 0.7^a*^	1.1 ± 0.1	242 ± 28^a**^
SS	386 ± 10	16.4 ± 0.7^a***b**^	1.3 ± 0.1^a*^	202 ± 41^a*^
Week 25				
CC	521 ± 13	10.4 ± 0.5	1.7 ± 0.1	81 ± 11
CS	578 ± 15^a,b**^	17.5 ± 1.2^a,b***^	2.7 ± 0.2^a***b**^	170 ± 22^a*^
SC	513 ± 7	10.7 ± 0.9	1.5 ± 0.1	104 ± 15
SS	574 ± 17^a,b**^	17.4 ± 1.2^a,b***^	2.8 ± 0.3^a,b***^	181 ± 37^a**b*^

Data are expressed as means ± SEM, *n* = 10, analysed by linear mixed models (lme), in R

*BW* body weight, *FFA* free fatty acids, *TG* triglyceride, *C* maternal Chow, *S* maternal chocolate and soft drink, *CC* maternal C–post-weaning C, *CS* maternal C–post-weaning S, SC maternal S–post-weaning C, SS maternal C–post-weaning S. C Chow diet, *S* Chow diet supplemented with chocolate and soft drink ad libitum

^a^Significant difference to CC. ^b^Significant difference to SC

*p  0.05, **p  0.01, ***p  0.001

### Hypothalamus anti-oxidative stress and inflammation

**Fig. 2:**
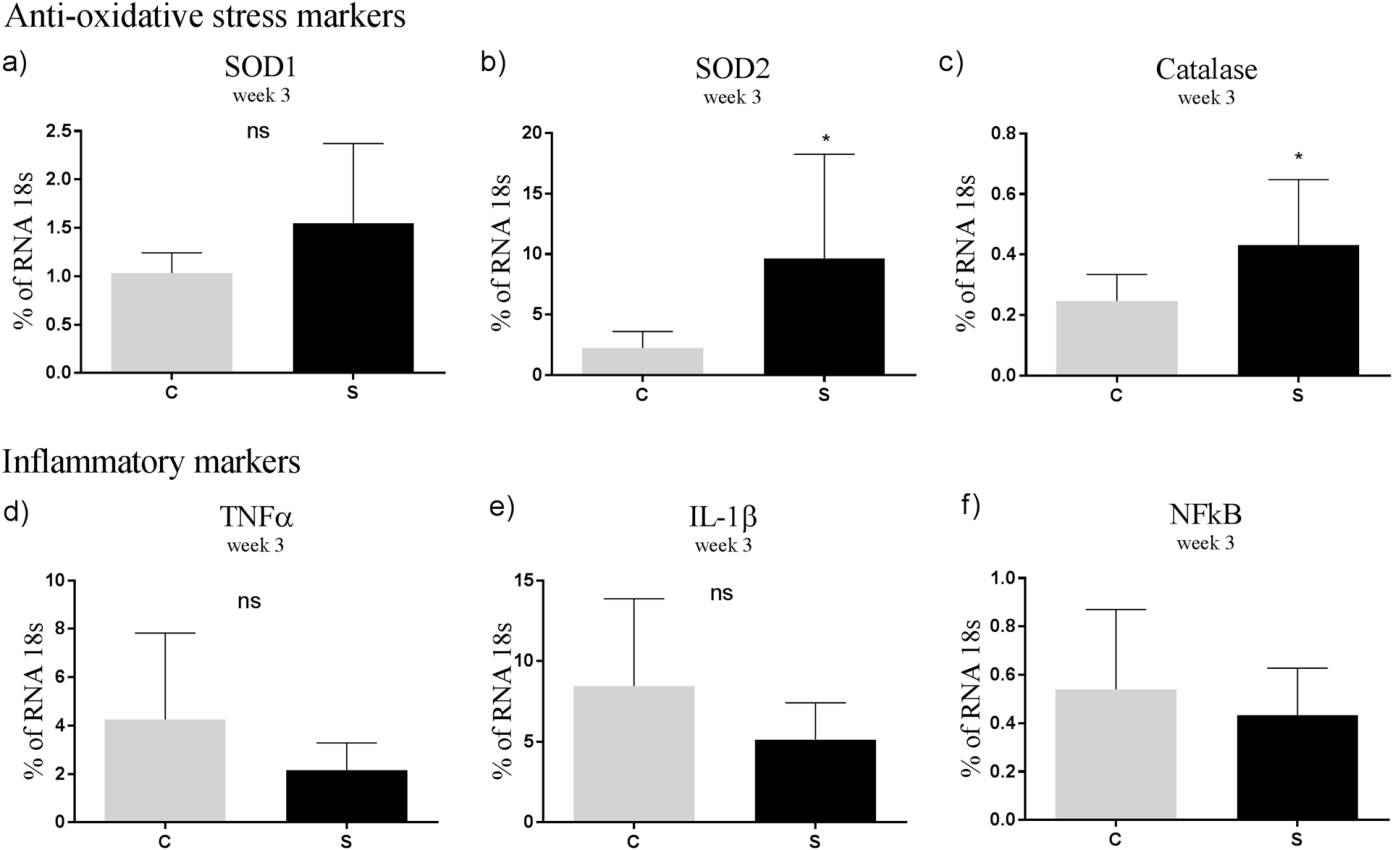
mRNA levels of hypothalamic oxidative stress and inflammatory markers in offspring aged 3 weeks. **a** SOD1, **b** SOD2, **c** catalase, **d** TNFα, **e** IL-1β, **f** NFκB. Data are expressed as means ± SEM, *n* = 8–10, analysed by mixed linear models (lme), unpaired *t*-test in R. (* *p* < 0.05, ** *p* < 0.01, *** *p* < 0.001). C Chow diet, S Chow diet supplemented with chocolate and soft drink ad libitum

**Fig. 3:**
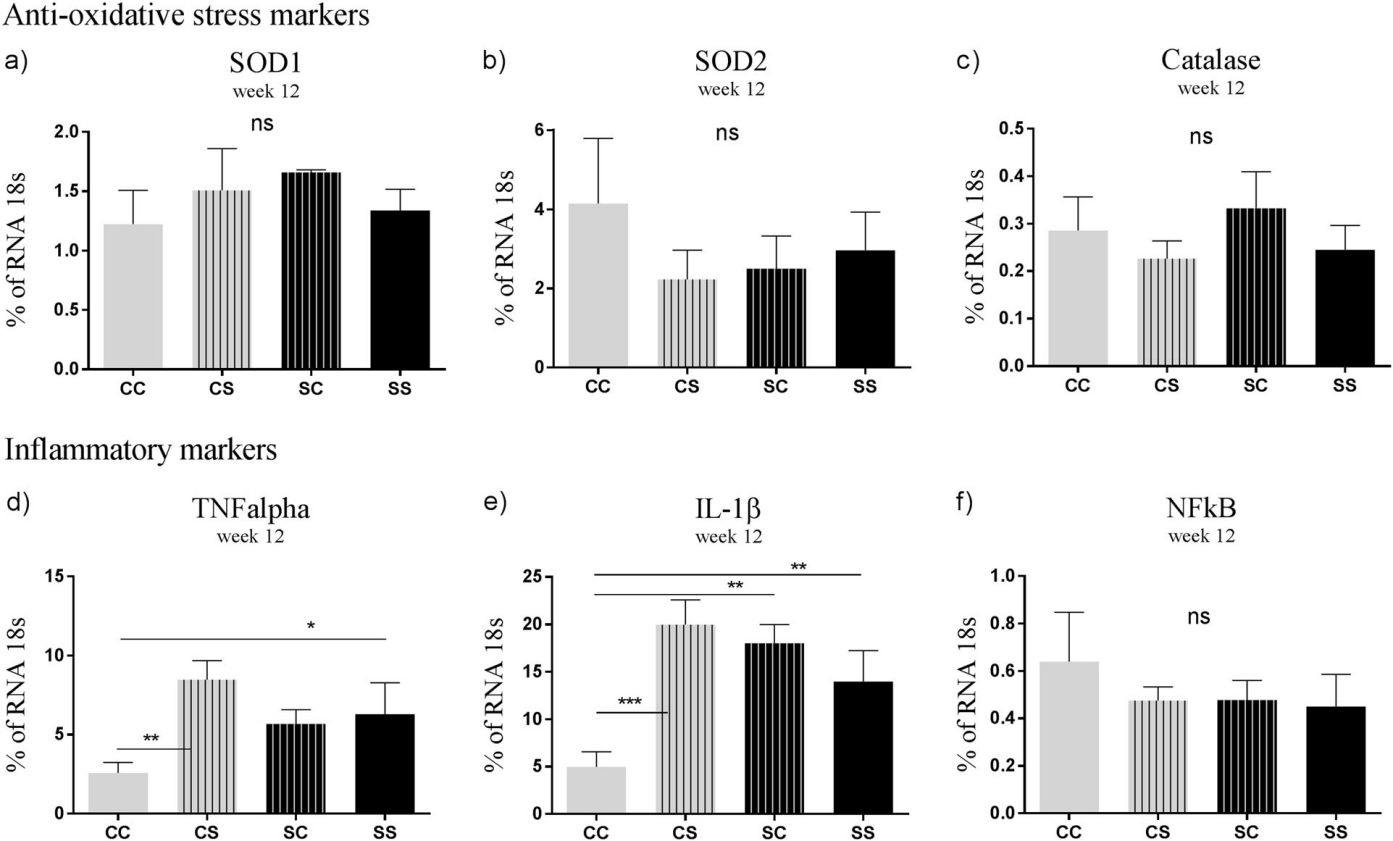
mRNA levels of hypothalamic inflammatory, microglial, astrocyte and oxidative stress markers in offspring aged 12 weeks. **a** SOD1, **b** SOD2, **c** catalase, **d** TNFα, **e** IL-1β, **f** NFκB. Data are expressed as means ± SEM, *n* = 8–10, analysed by linear mixed models (lme), unpaired *t*-test in R. (*p < 0.05, **p < 0.01, ***p < 0.001). CC maternal C–post-weaning C, CS maternal C–post-weaning S, SC maternal S–post-weaning C, SS maternal C–post-weaning S. C Chow diet, S Chow diet supplemented with chocolate and soft drink ad libitum

At 3 weeks of age, offspring from S fed mothers had increased mRNA levels of the hypothalamic anti-oxidative markers SOD2 (*p* < 0.05) and catalase (*p* < 0.05) compared to offspring from mothers fed the chow diet. Moreover, the mRNA level of SOD1 was slightly increased in S offspring compared to C offspring, but failed to reach statistical significance (*p* = 0.08). No significant difference was observed in the inflammatory markers between the groups (Fig. 2). At 12 weeks of age, the mRNA levels of hypothalamic anti-oxidative markers did not differ between the groups. However, mRNA levels of the pro-inflammatory markers TNF-α and IL-1β were increased in CS and SS offspring fed postnatal S diet compared to CC offspring (*p* < 0.01, *p* < 0.05). The mRNA level of IL-1β was significantly increased in CS, SC and SS offspring compared to CC offspring (*p* < 0.01, *p* < 0.001) (Fig. 3).

### Glucose homeostasis and lipid plasma profile

**Fig. 4:**
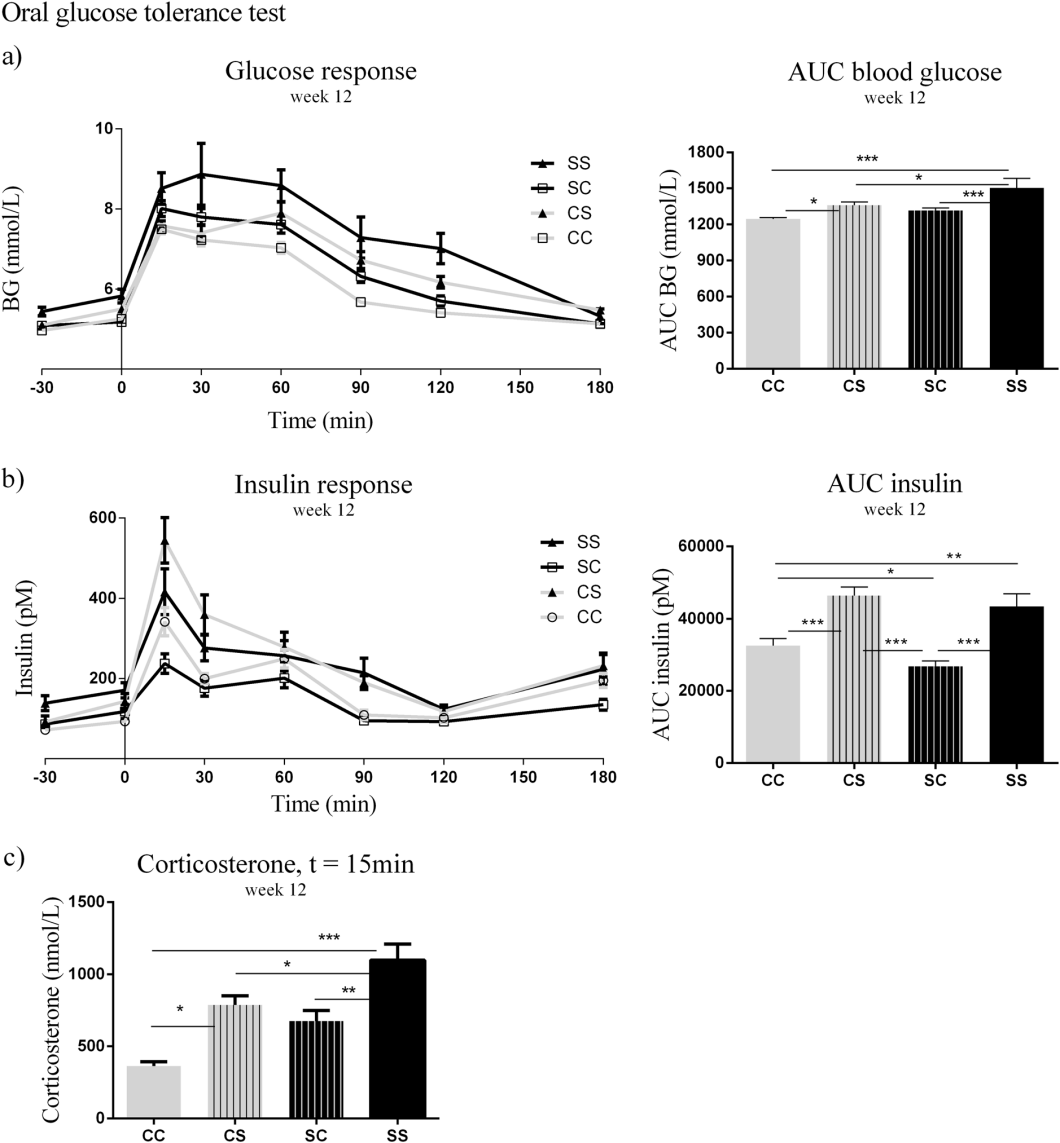
Metabolic plasma profiles of offspring aged 12 weeks. **a** OGGT glucose response, **b** OGGT insulin response, **c** OGGT, corticosterone. Data are expressed as means ± SEM, *n* = 8–10, analysed by linear mixed models (lme), unpaired t-test in R. (*p < 0.05, **p < 0.01, ***p < 0.001). CC: maternal C–post-weaning C, CS maternal C–post-weaning S, SC maternal S–post-weaning C, SS maternal C–post-weaning S, C Chow diet, S Chow diet supplemented with chocolate and soft drink ad libitum

At the oral glucose tolerance test (OGTT) at 12 weeks of age, the area under the curve (AUC) for blood glucose was larger in CS and SS offspring than in CC offspring (*p* < 0.05, *p* < 0.001). Furthermore, SS offspring had increased AUC of blood glucose compared to SC (*p* < 0.001) and CS (*p* < 0.05) (Fig. 4a). AUC of plasma insulin was increased in both CS and SS offspring compared to SC (*p* < 0.001) and CC offspring (*p* < 0.001, *p* < 0.01). We also observed that SC offspring had a reduced AUC of plasma insulin compared to CC (*p* < 0.05) (Fig. 4b). During the OGTT, we measured the levels of the stress hormone corticosterone 15 min after the glucose load. SS offspring had increased plasma corticosterone levels compared to SC (*p* < 0.01), CS (*p* < 0.05) and CC (0.001). CS offspring also had higher levels of corticosterone compared to CC offspring (*p* < 0.05) (Fig. 4c).

**Fig. 5:**
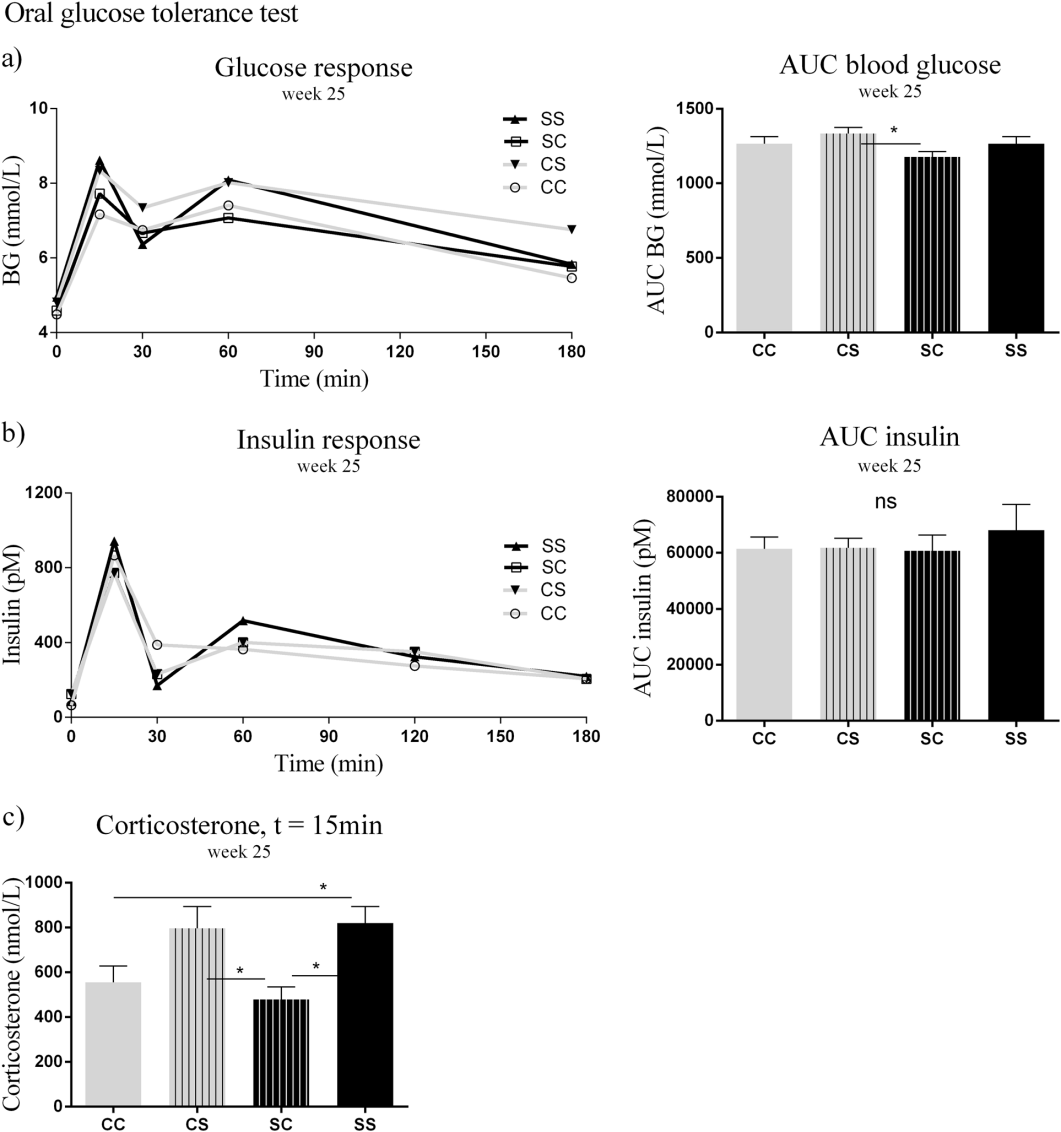
Metabolic profile in plasma of offspring aged 25 weeks. **a** OGGT glucose response, **b** OGGT insulin response, **c** OGGT, corticosterone. Data are expressed as means ± SEM, *n* = 8–10, analysed by linear mixed models (lme), unpaired *t*-test in R. (*p < 0.05, **p < 0.01, ***p < 0.001). CC maternal C–post-weaning C, CS maternal C–post-weaning S, SC maternal S–post-weaning C, SS maternal C–post-weaning S, C Chow diet, S Chow diet supplemented with chocolate and soft drink ad libitum

The glucose tolerance at 25 weeks of age showed that CS offspring had significantly higher AUC of blood glucose compared to SC offspring (*p* < 0.05) (Fig. 5a). No significant differences were observed in AUC of plasma insulin between the groups (Fig. 5b). The corticosterone levels showed that SS offspring had higher response than SC (*p* < 0.05) and CC (*p* < 0.05), while CS had higher corticosterone levels than SC (*p* < 0.05) (Fig. 5c).

## Discussion

The hypothalamus is suggested to be one of the first tissues to show signs of inflammation upon HFD exposure predisposing to obesity^12,19,33,34^. The present study supported the hypothesis that early changes in the hypothalamus can predispose for obesity, as the hypothalamic inflammatory response preceded increased weight gain^32^. Only limited focus has been given to hypothalamic oxidative stress and inflammation in recent publications on metabolic programming. We demonstrated that offspring exposed to maternal chocolate and soft drink (S) had increased early anti-oxidative markers, such as SOD2 and catalase, which is likely a response to protect against cellular reactive oxidative stress (ROS) and damage^23^. However, at 12 weeks of age expression of the anti-oxidative markers tended to decrease, which may indicate a reduced ability to eliminate ROS. At the same time, pro-inflammatory markers, such as TNFα and IL-1β, were up-regulated in the hypothalamus of all offspring exposed to the S diet during some point of their life (more pronounced in the offspring fed with postnatal S diet). Hypothalamic expression of NFκB was not affected by the increase of TNFα and IL-1β as previously found^18,28,35,36^. However, since astrogliosis was observed in the adult offspring fed with postnatal S diet^32^, increased activation of the NFκB pathway may likely appear later as JNK and NF-κB pathways often drive development of astrogliosis^37,38^. Nevertheless, the study results indicated that hypothalamic oxidative stress likely appears before inflammation and the rise in anti-oxidative stress response could not counteract hypothalamic inflammation in the offspring exposed to the maternal S diet. In addtition to hypothalamic inflammation in the offspring aged 12 weeks, the increased adiposity and plasma FFA may also affect hypothalamic neuronal circuits through increased systemic cytokines and adipokines and contributing to the obese phenotype^12,39–41^. At 12 weeks of age, hypothalamic inflammation was not associated with impaired glucose tolerance or insulin resistance as previously indicated^15–19^. Impaired glucose tolerance was only observed in CS and SS offspring and not in SC offspring at 12 weeks of age. Also, no indications of impaired glucose tolerance was observed at 25 weeks of age, although CS and SS offspring had developed astrogliosis^32^. Thus, neuronal areas responsible for peripheral glucose homeostasis was not affected by the intake of S diet at 25 weeks of age. The impaired glucose tolerance at 12 weeks of age could rather be an effect of reduced insulin sensitivity as insulin secretion was increased. The increased insulin secretion may be a result of increased β-cell mass as this has been observed in rats fed with a candy supplement^42^. At 25 weeks of age, a higher glucose bolus (4 mg/kg) was used in the OGTT to ensure a sufficient glucose response and to obtain a clear difference in insulin secretion in the offspring. The findings revealed that offspring in every group had an increase in insulin capacity to lower the glucose bolus. This was indicated by a higher insulin peak and same glucose profile compared to the 12 weeks offspring, suggesting that the glucose handling was not decreased at 25 weeks of age but rather reversible. During the OGTT, the glucose tolerance and insulin secretion in CS and SS offspring were comparable to CC offspring despite obesity, suggesting a compensatory response compared to the findings at 12 weeks of age. This may either have occurred as a result of up-regulated peripheral insulin sensitivity or increased pancreatic insulin secretory capacity. It has been reviewed previously that maternal HFD was associated with impaired glucose regulation in the offspring at different ages^43–46^, but long-term impaired glucose tolerance or insulin resistance has not been observed (25 weeks of age)^46^ resembling the results in the present study. Furthermore, Fernandez-Twinn and colleagues demonstrated that offspring exposed to a maternal low-protein diet developed improved glucose tolerance in young adulthood (20 weeks of age) followed by an age-dependent loss of glucose tolerance at 21 months of age^47^. This could also be true for offspring exposed to the maternal HFD^46^ or maternal S diet in the present study. In addition, during the OGTT, increased plasma corticosterone levels were observed 15 min after glucose bolus in CS and SS offspring at both 12 and 25 weeks of age suggesting that offspring exposed to postnatal S were more stress-sensitive; however, it did not have an effect on glucose tolerance at 25 weeks of age. Also, in the litterature, there are generally conflicting data in both obese humans and rodent models of obesity, as to whether obesity is associated with an increased stress sensitivity^48^.

Interestingly, SC offspring fed chow diet after weaning was not able to prevent the increase in hypothalamic inflammatory response, adiposity and FFA at 12 weeks. This is consistent with a study of HFD-fed rats that were switched back to a standard chow diet for 8 weeks. These rats lost their excess weight, whereas they maintained an increase in hypothalamic inflammatory markers^49^. However, it cannot be ruled out that a hypothalamic inflammatory state may be reversible in HFD-fed rats over a longer period of time with standard chow diet than 8–9 weeks. In the present study, adiposity and plasma lipids were normalized, and no signs of astrogliosis was found, in SC offspring at 26 weeks of age, indicating that metabolic changes induced by maternal S diet could apparently be reversed by exposure to a healthy nutrient after weaning, which is in agreement with previous findings^50–53^. Nevertheless, despite normalization of the metabolic profile, SC offspring may be prone to develop metabolic disorders later in life compared to offspring exposed to a maternal healthy diet, as early metabolic changes may have long-lasting effects^54,55^.

In conclusion, we demonstrated that indications of hypothalamic oxidative stress was observed prior to the inflammatory response in offspring exposed to a maternal S diet. Furthermore, both maternal and postnatal S induced hypothalamic inflammation at 12 weeks of age associated with increased adiposity and plasma FFA. This metabolic profile appeared prior to weight gain, thus contributing to the obese phenotype in adulthood. Hypothalamic inflammation was not reversed at 12 weeks of age by switching to chow after weaning, indicating an increased sensitivity to hypothalamic changes in response to maternal S. No correlation was found between hypothalamic inflammation and impaired glucose tolerance and insulin secretion. At 12 weeks of age, reduced glucose tolerance was found in offspring exposed to the postnatal S diet; however, this was reversed at 25 weeks of age, which points towards a compensatory response of the insulin sensitivity or insulin secretion.
